# Enhanced Cellular Uptake and Photodynamic Effect with Amphiphilic Fluorinated Porphyrins: The Role of Sulfoester Groups and the Nature of Reactive Oxygen Species

**DOI:** 10.3390/ijms21082786

**Published:** 2020-04-16

**Authors:** Barbara Pucelik, Adam Sułek, Agnieszka Drozd, Grażyna Stochel, Mariette M. Pereira, Sara M. A. Pinto, Luis G. Arnaut, Janusz M. Dąbrowski

**Affiliations:** 1 Faculty of Chemistry, Jagiellonian University, 30-387 Krakow, Poland; 2 Małopolska Center of Biotechnology, Jagiellonian University, 30-387 Krakow, Poland; 3 Chemistry Department, University of Coimbra, 3004-535 Coimbra, Portugal

**Keywords:** anticancer activity, photodynamic therapy, porphyrins, photosensitizers, reactive oxygen species, singlet oxygen

## Abstract

A class of amphiphilic photosensitizers for photodynamic therapy (PDT) was developed. Sulfonate esters of modified porphyrins bearing—F substituents in the *ortho* positions of the phenyl rings have adequate properties for PDT, including absorption in the red, increased cellular uptake, favorable intracellular localization, low cytotoxicity, and high phototoxicity against A549 (human lung adenocarcinoma) and CT26 (murine colon carcinoma) cells. Moreover, the role of type I and type II photochemical processes was assessed by fluorescent probes specific for various reactive oxygen species (ROS). The photodynamic effect is improved not only by enhanced cellular uptake but also by the high generation of both singlet oxygen and oxygen-centered radicals. All of the presented results support the idea that the rational design of photosensitizers for PDT can be further improved by better understanding the determinants affecting its therapeutic efficiency and explain how smart structural modifications can make them suitable photosensitizers for application in PDT.

## 1. Introduction

Photodynamic therapy (PDT) employs a photoactive molecule named a photosensitizer (PS), light absorbed by the PS and the molecular oxygen present in tissues to generate reactive oxygen species (ROS) [1]. ROS, such as singlet oxygen, superoxide ion, or hydroxyl radical, lead to the oxidation of biologically relevant molecules and cause irreversible destruction of target tissues by cell death, vascular damage, and inflammation. Compared to traditional treatments (surgery, radiotherapy, and chemotherapy), PDT is an effective and safe method of cancer treatment with minimal impact on surrounding healthy tissue. Moreover, PDT leads to the stimulation of antitumor immune response, which may allow eliminating not only primary tumors but also protecting against metastases [2,3,4]. A major challenge in PDT is to develop PS molecules that absorb light in the phototherapeutic window—the 630–850 nm range—where human tissues are the most transparent. Porphyrin derivatives [5], including chlorins [6,7,8] and bacteriochlorins [6], as well as phthalocyanines [9,10,11], are the most frequently studied PDT photosensitizers due to their long-wavelength absorption and their photophysical properties (high yields of long-lived triplet states) [12,13,14,15]. The lowest-electronically excited triplet state of photosensitizer is the precursor of ROS-generating reactions. ROS may be generated by transferring an electron or hydrogen atom (type I processes) or electronic energy (type II processes) to molecular oxygen, with the formation of oxygen-centered radicals (superoxide ion and hydroxyl radicals) and singlet oxygen, respectively.

Various tetrapyrrolic-based photosensitizers have been approved by the U.S. Food and Drug Administration (FDA) and have entered clinical practice such as porfimer sodium (Photofrin^®^), verteporfin (Visudyne^®^), temoporfin (Foscan^®^), and more recently padeliporfin (Tookad Soluble^®^). Nevertheless, there are still significant challenges in the development of photosensitizers that have specificity to tumors cells and adequate clearance from healthy cells. Increased lipophilicity usually imparts better cellular uptake but may lead to aggregation and decreased ROS generation. Too much hydrophilicity diminishes active transport across the cell membrane, [16] and limits the use of the PS to vascular-targeted PDT. Balancing the lipophilic/hydrophilic character of PS is the yin and yang of cellular uptake and PDT efficacy [4,17]. Various nanoscale drug carriers, such as polymer-based nanoparticles [18,19], liposomes [20], and polymeric micelles [21,22], have been explored as delivery systems for both hydrophilic and hydrophobic photosensitizers [23,24,25,26]. The polarity and geometry of substituents around the macrocycle offer decisive control over the amphiphilic character of the PS with a significant impact on cellular uptake and subcellular localization [27]. Amphiphilic photosensitizers may be designed to combine increased tumor uptake and rapid systemic clearance [28].

We showed that halogenated and sulfonated porphyrins are simple to synthesize, have photophysical properties appropriate for PDT, and lead to a photodynamic effect comparable to that of Photofrin [29]. Subsequently, it was demonstrated that bacteriochlorins (e.g. sulfonamide derivatives bearing Cl or F substituents in the *ortho* positions of the phenyl rings) have even better properties for PDT, including strong absorption in the near-infrared (λ_max_ ≈ 750 nm, ε ≈ 10^5^ M^−1^cm^−1^), excellent cellular uptake, intracellular localization, low cytotoxicity in the dark, and high phototoxicity upon irradiation with red light [6,28,30,31,32,33]. These and other bacteriochlorin-based photosensitizers proved to be very efficient in vascular-targeted PDT [34,35]. One of these photosensitizers, named redaporfin, is currently in clinical trials [36]. Following a different strategy, we reported a simple and efficient synthetic method to tune the amphiphilicity of sulfonate ester porphyrins by selecting the type and number of fluorine atoms as well as the length of the alkyl sulfonate ester chains [37,38]. It is recognized that sulfonic acid esters are relatively unstable since they may react with nucleophiles *in vitro* and *in vivo* and possibly hydrolyze to sulfonate. Sulfonic acid esters can act as chemotherapeutics due to their alkylating properties (*e.g*., busulfan) [39]. However, the stability of sulfonic acid esters greatly depends on the substitution pattern. Very stable esters can be obtained if suitable, electron-withdrawing, deactivating substituents are introduced [40,41].

**Scheme 1:**
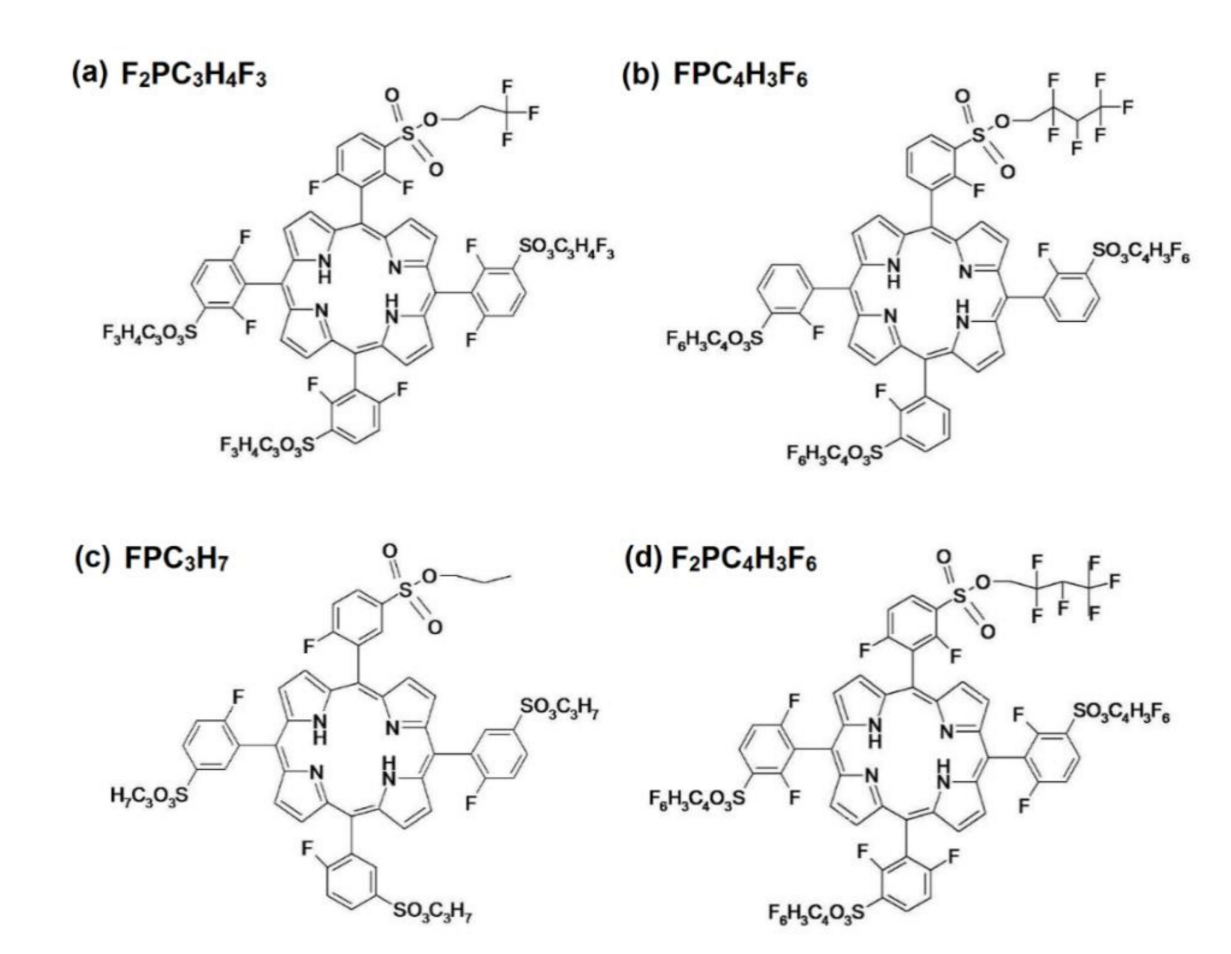
Chemical structures of fluorinated sulfoester porphyrins studied in this work.

Sulfonate ester porphyrins bearing fluorine atoms are an interesting template to explore photosensitizers with polarities that may change after administration in a controlled manner. In the past, we explored halogenated and sulfonated porphyrins to assess the potential of this molecular template before preparing the corresponding bacteriochlorin, namely redaporfin. Herein, we characterize a series of amphiphilic polyfluorinated sulfonate ester porphyrins with four different substitution patterns in terms of their photochemistry, cellular uptake, subcellular localization, dark cytotoxicity, and photodynamic effect. We show that sulfonate ester porphyrin derivatives offer new leads for PDT photosensitizers with appropriate amphiphilicity and high phototoxicity. The chemical structures of investigated porphyrins are presented in Scheme 1.

## 2. Results and Discussion 

### 2.1. Photosensitizers and Their Optical Properties

The synthesis of *meso*(sulfonate ester fluoroaryl)porphyrins has been previously described [38]. In order to synthesize the desired fluorinated amphiphilic porphyrins, containing sulfonate ester appendices, the derivatization of 5,10,15,20-tetrakis(2-fluorophenyl)porphyrin and 5,10,15,20-tetrakis(2,6-difluorophenyl)porphyrin into the corresponding chlorosulfonated porphyrins was achieved by mixing the porphyrins with an excess of chlorosulfonic acid. The chlorosulfonic acid group is susceptible to react with alcohols in basic conditions. This synthetic strategy allowed us not only to prepare a family of amphiphilic porphyrins, containing several sulfonate ester appendices, but also the inclusion of one or more fluorine atoms in the porphyrin structure [38]. 

**Figure 1:**
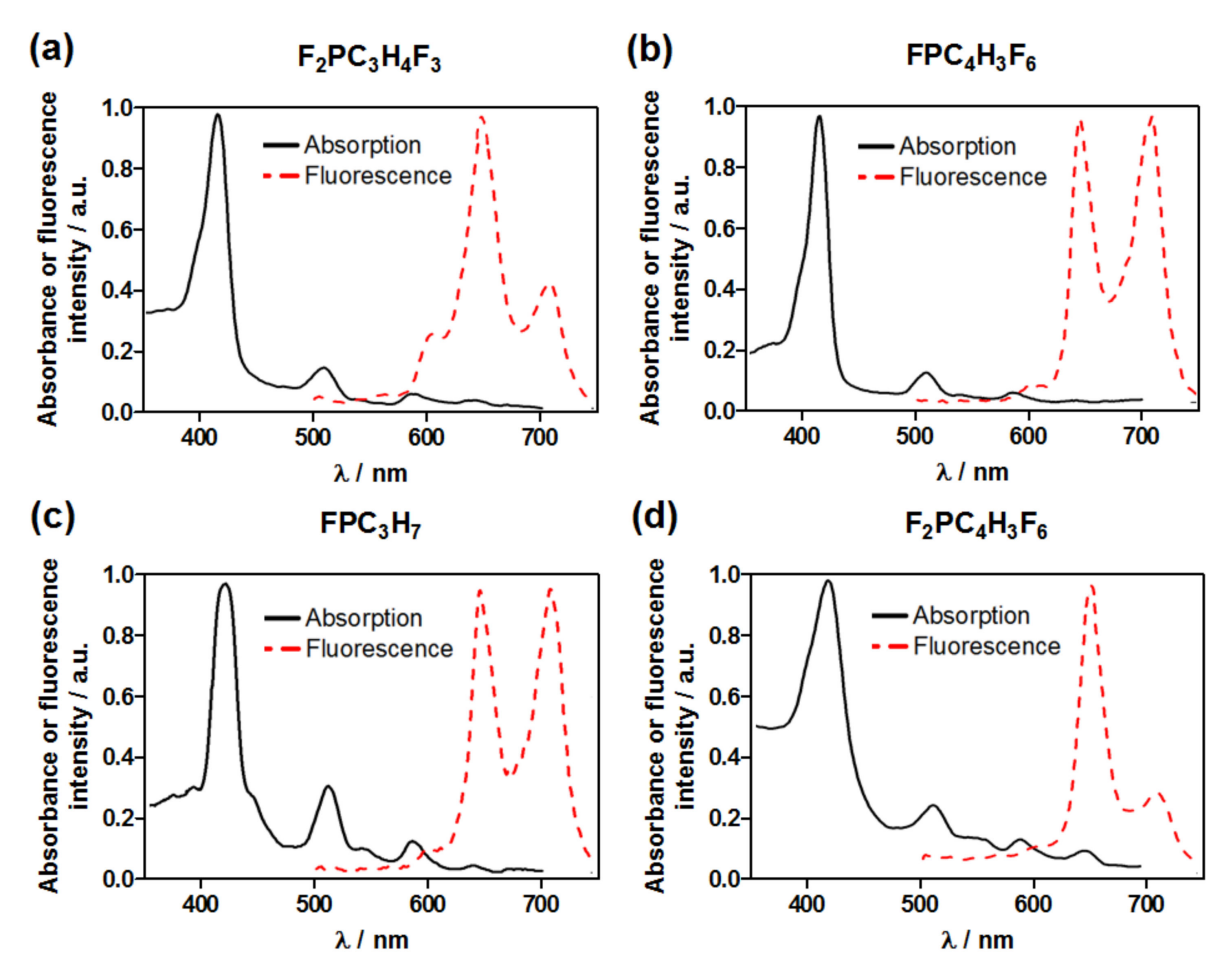
UV-VIS-NIR electronic absorption and fluorescence (dashed) spectra of investigated porphyrins: (**a**) F_2_PC_3_H_4_F_3_, (**b**) FPC_4_H_3_F_6_, (**c**) FPC_3_H_7_, (**d**) F_2_PC_4_H_3_F_6_ registered in DMSO at room temperature.

Some of us reported the fundamental spectroscopic and photophysical properties of investigated photosensitizers determined in ethanol [31,38]. The ground state absorption and fluorescence spectra of sulfonate ester porphyrin derivatives, recorded at room temperature in DMSO, are presented in Figure 1. The spectra of all porphyrins show the characteristic bands originated from free base porphyrin with the D_2h_ symmetry. While symmetry dictates the presence of distinct B_x_ and B_y_ transitions, a single Soret band is observed at ~420 nm in the experimental spectrum, which is typical for free-base porphyrins. An interesting feature that is a fingerprint of free-base porphyrins is the presence of four Q bands in the visible region. These extra bands are due to vibrational coupling effects, namely vibronic structures, and derive from the break of the degeneration of the LUMOs b_2g_ and b_3g_ introduced by the presence of NH central bonds [42,43]. 

Their spectra can be interpreted in terms of the four orbital Gouterman’s model, where the principal excitations involve the two highest occupied molecular orbitals (HOMO and HOMO-1) and the two lowest unoccupied molecular orbitals (LUMO and LUMO+1). S_1_ and S_2_ are mixing both H→L and H→L+1 orbitals and may even include other small contributions, which was confirmed by our results. The calculations of theoretical spectra presented in Table S1 and Table S2 are in line with this assignment. The comparison with experimental UV-Vis-NIR electronic absorption spectra registered in DMSO reveals that the theoretical energies of the transitions are ca. 0.1 eV higher than the experimental values, and up to 0.3 eV higher for the transition of the lowest energy. The isodensity contour plots are centered on the porphyrin ring without a significant contribution from the fluorinated substituents and confirmed that they all have a π−›π* character (Figure S1).

The longest-wavelength absorption band at 635–645 nm is crucial for PDT application, because only red and NIR light has sufficient tissue penetration ability. The fluorescence excitation spectra of all the compounds are typical and correspond well with their absorption spectra. Similar spectroscopic properties characterize all of the studied porphyrins. However, according to our previous data published for sulfonic derivative (F_2_POH), some of them (especially difluorinated ones) are characterized by a more intense Q_x_ band, which is further displaced to the red region of spectra (close to 650 nm). Considering that F_2_POH was shown to be efficient photosensitizer in vitro in both anticancer photodynamic effect as well as antimicrobial photoinactivation [31], it can be expected that the sulfonate ester substituents studied in this work may increase the phototoxicity of these photosensitizers.

### 2.2. Detection of Reactive Oxygen Species Using Fluorescent Probes

**Figure 2:**
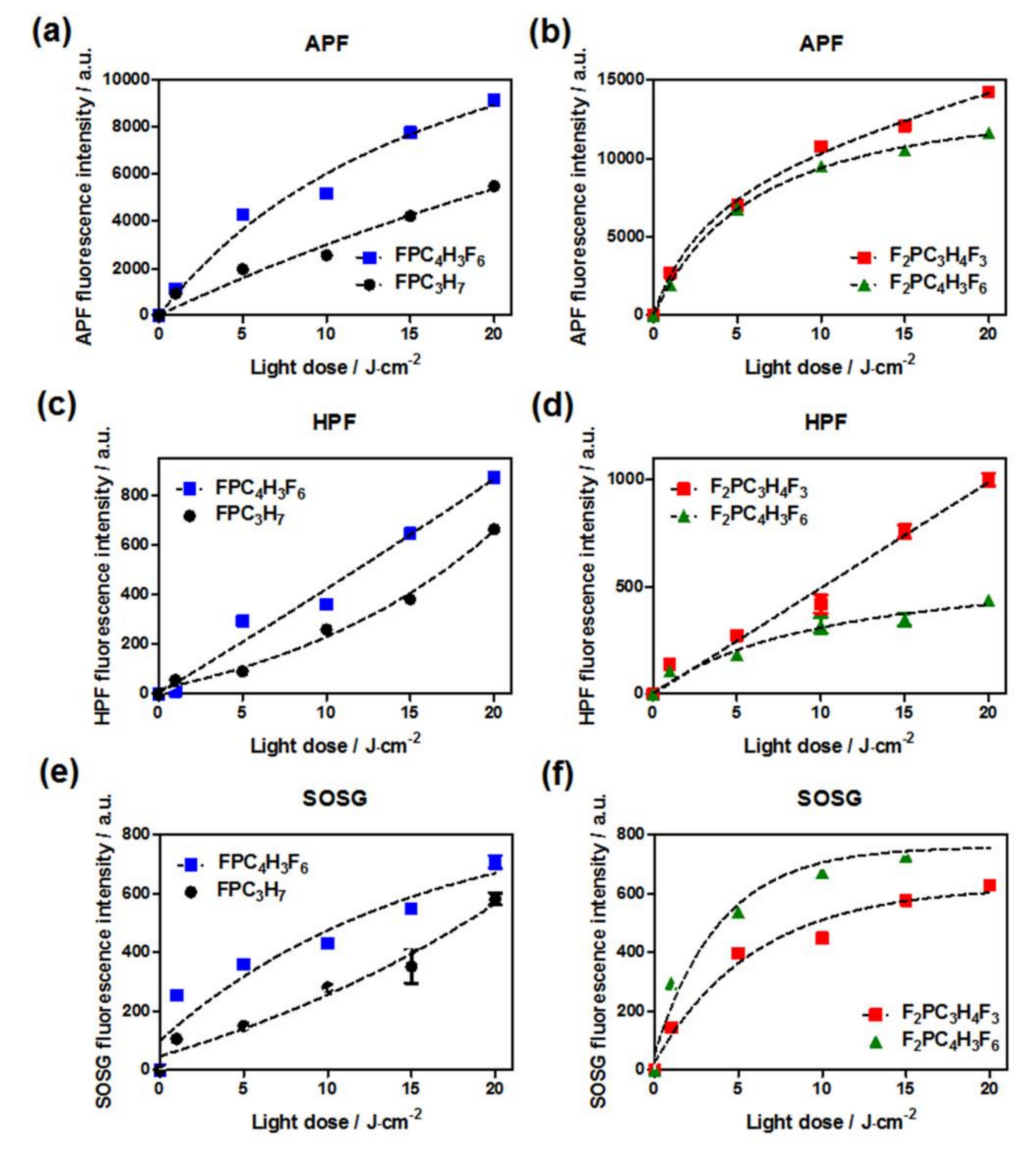
Detection of photogenerated reactive oxygen species using fluorescent probes at a concentration of 15 µM: APF (**a**,**b**), HPF (**c**,**d**), SOSG (**e**,**f**); during irradiation of photosensitizer solution (10 µM).

Considering the potential applications of investigated porphyrins, their ability to generate ROS via type I (hydrogen or electron transfer) or type II (energy transfer) photochemical reactions were determined using fluorescent probes selective or specific for each species (APF, HPF, SOSG), Figure 2. As indicated, even though ^1^O_2_ (^1^Δ_g_) is thought to be the dominant cytotoxic ROS in porphyrin-mediated PDT, other types of ROS can also be generated through photoinduced electron transfer reaction. The Φ_Δ_ values are strongly affected by the substitution pattern and reached the highest values for polyfluorinated sulfonate ester 0.81 ± 0.02 for FPC_4_H_3_F_6_, 1.0 ± 0.04 for F_2_PC_4_F_3_F_6_ and relatively lower for porphyrins with reduced fluorine atoms in alkoxy chain F_2_PC_3_H_4_F_3_ and FPC_3_H_7_ (0.74) [38]. The significantly higher Φ_Δ_ values can be related to the presence of many electron-withdrawing groups and heavy atom effect in the PS structure leading to higher ^1^O_2_ generation, reflecting an optimized triplet energy transfer from these compounds to molecular oxygen.

The singlet oxygen quantum yield determined for fluorinated porphyrin with the highest number of halogenated atoms in alkoxy chain (Φ_Δ_ = 1.0 ± 0.04 for F_2_PC_4_H_3_F_6_) is significantly higher than those observed for the porphyrin with nonfluorinated sulfoester substituents (Φ_Δ_ = 0.74 ± 0.02 for FPC_3_H_7_, respectively) [38]. These results are in agreement with those obtained with SOSG, which also indicated that polyfluorinated sulfonate ester derivative is a relatively better singlet oxygen generator (Figure 2). This effect can be related to the nature of each substituent, because halogen atoms enhance the heavy atom effect due to spin-orbital coupling, and consequently, promote the singlet oxygen quantum yields [44,45]. On the other hand, the competitive type I mechanism may occur and also play an important role in PDT. To confirm the presence of type I reaction products, we applied APF and HPF probes. HPF was reported as selective and specific for ^•^OH, whereas APF can also be sensitive to other radical species [46]. As shown in Figure 2, the increase in fluorescence signal derived from each probe can be observed with increased light dose. Hence, both type I and type II mechanisms may occur competitively, and their relative contributions depend on the photosensitizer, substrates, and environment [47].

It should be noted that we used two light sources for selected porphyrin due to their differences in the absorption maximum in the phototherapeutic window. The application of the 635 nm emitting diode and 655 nm diode laser light allow us to excite the compounds with appropriate wavelengths and then determined applied light doses. Therefore, a more direct comparison between all the photosensitizers was possible. The same light source and experimental conditions were employed for each photosensitizer in *in vitro* photodynamic activity evaluation.

### 2.3. Lipophilicity of Photosensitizer (logP Determination)

Lipophilicity of photosensitizers was estimated by the logarithm of a partition coefficient, log*P*, which reflects the equilibrium partitioning of a molecule between a nonpolar and a polar phase, such as an n-octanol/PBS system. Partition coefficients were obtained experimentally by the modified shake-flask method and are presented in Table 1 in terms of their logarithmic values (log*P*). These results confirm the hydrophilicity of sulfonate derivative (F_2_POH), whereas the data obtained from a set of sulfonate ester porphyrin revealed the increased, but similar lipophilicity (log*P* 1.4–1.6). The results indicate that besides the overall amphiphilicity, the presence of sulfonate ester in a molecule effectively increases log*P* value as compared with hydrophilic, sulfonic analog F_2_POH (log*P* = –1.7). This effect plays an important role in their cellular uptake by cancer cells, tumor selectivity, and overall PDT efficacy [30,48,49].

### 2.4. Biological Studies

#### 2.4.1. Cellular Uptake

**Figure 3:**
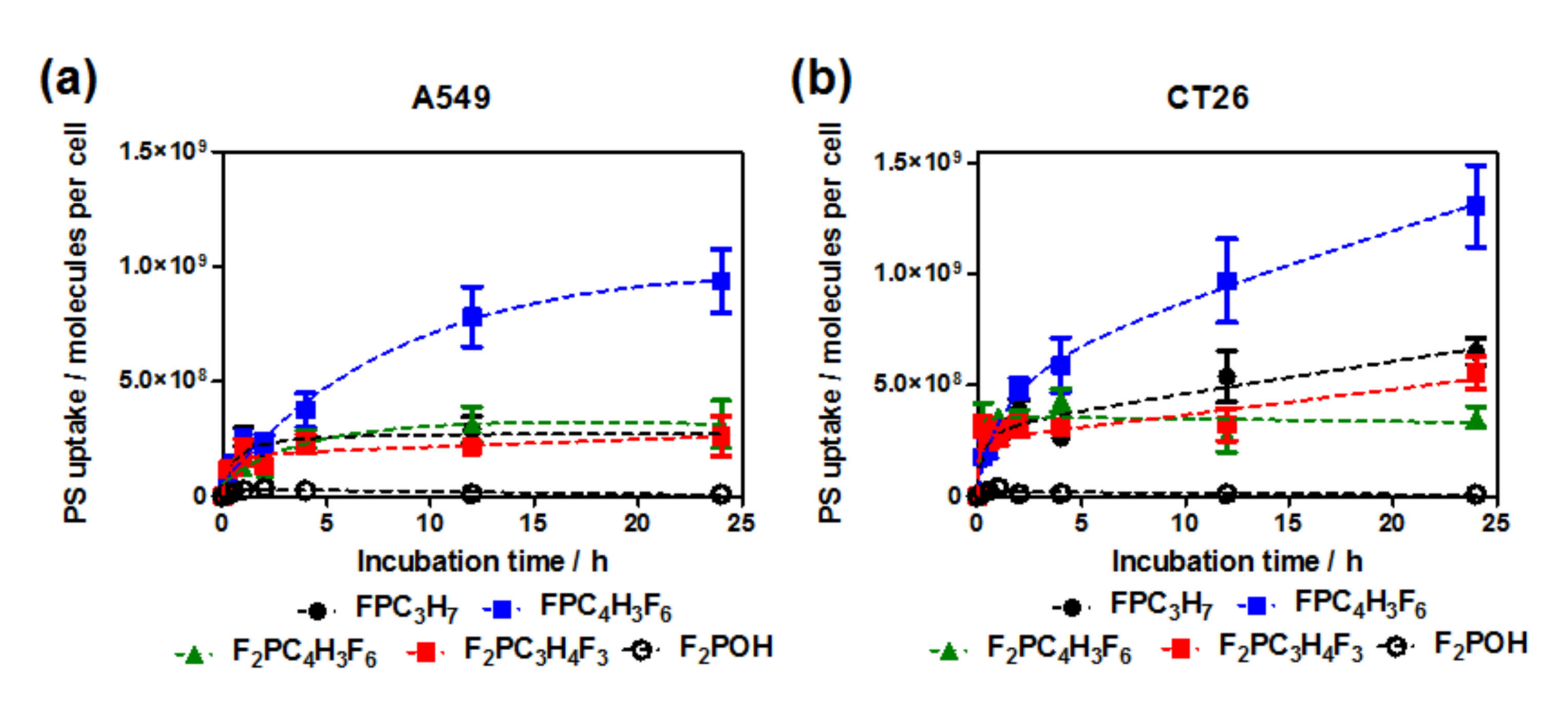
Time-dependent cellular uptake of investigated porphyrins at 20 μM by (**a**) A549 and (**b**) CT26 cells determined by fluorescence intensity measurements in cellular extracts of each compound.

**Figure 4:**
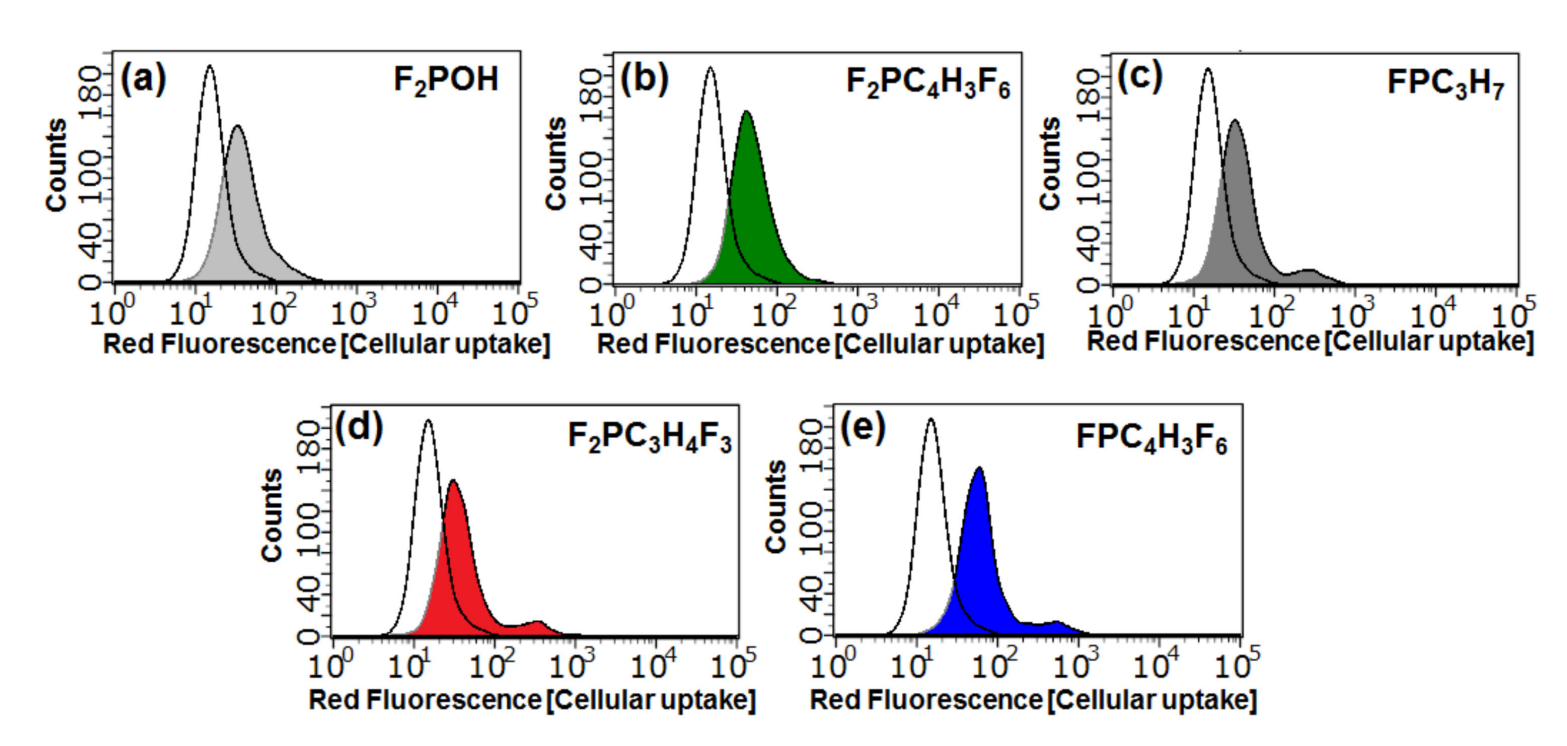
Cellular uptake determined in CT26 cells based on the red fluorescence of each porphyrin using flow cytometry. Histograms of the fluorescence intensities of the photosensitizer in CT26 (colored) or untreated cells (gray): (**a**) F_2_POH, (**b**) F_2_PC_4_H_3_F_6_, (**c**) FPC_3_H_7_, (**d**) F_2_PC_3_H_4_F_3_, (**e**) FPC_4_H_3_F_6_. The cells (0.5×10^6^ cells/mL) were incubated with 20 µM PS for 24 h, then washed twice with HBSS and analyzed with flow cytometry.

The time-dependent accumulation of sulfonate ester porphyrins and their sulfonic analog in A549 and CT26 cells exposed to 5 μM photosensitizer concentration is shown in Figure 3. The data indicate enhanced cellular uptake in both cell lines for sulfonate ester derivatives. In A549 cells, the uptake stabilizes after 12 h of incubation, while the accumulation in CT26 continues to increase for a longer time. After 24 h of incubation the following orders of increasing accumulation were found: FPC_4_H_3_F_6_ > F_2_PC_4_H_3_F_6_ ≈ F_2_PC_3_H_4_F_3_ ≈ FPC_3_H_7_ >> F_2_POH for A549 cells and FPC_4_H_3_F_6_ > FPC_3_H_7_ > F_2_PC_3_H_4_F_3_ > F_2_PC_4_H_3_F_6_ >> F_2_POH for CT26. We also confirmed those trends in CT26 cells by performing flow cytometry analysis that also indicated the most effective cellular uptake for FPC_4_H_3_F_6_, Figure 4.

In all cases, the highest uptake was observed for the sulfonate ester photosensitizers and the lowest for the sulfonated one. The replacement of the sulfonic groups, which are ionized in PBS, by the sulfonate ester groups appreciably enhances cellular uptake. Under these conditions, the chromophore of sulfonate ester porphyrins seems to be stable. Their higher cell uptake is consistent with the higher cell membrane permeation of neutral and amphiphilic drugs. They seem to combine enough lipophilicity to increase the affinity toward cells but not as much as to aggregate extensively in an aqueous culture medium. Based on the singlet oxygen quantum yields and the amount of photosensitizer present in the cells, it can be expected that sulfonate ester derivatives are interesting photosensitizers for PDT.

#### 2.4.2. CLSM Imaging and Subcellular Localization

**Figure 5:**
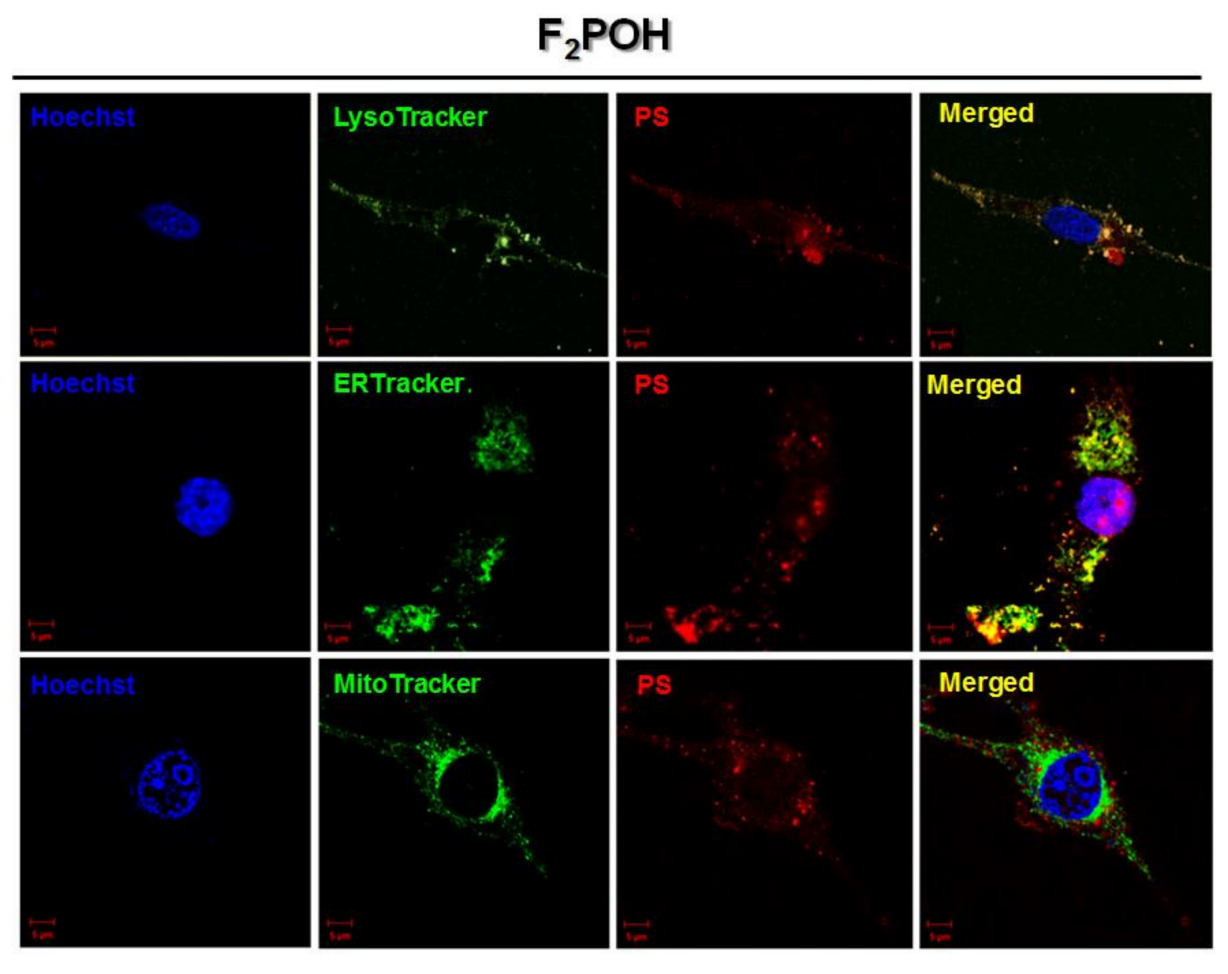

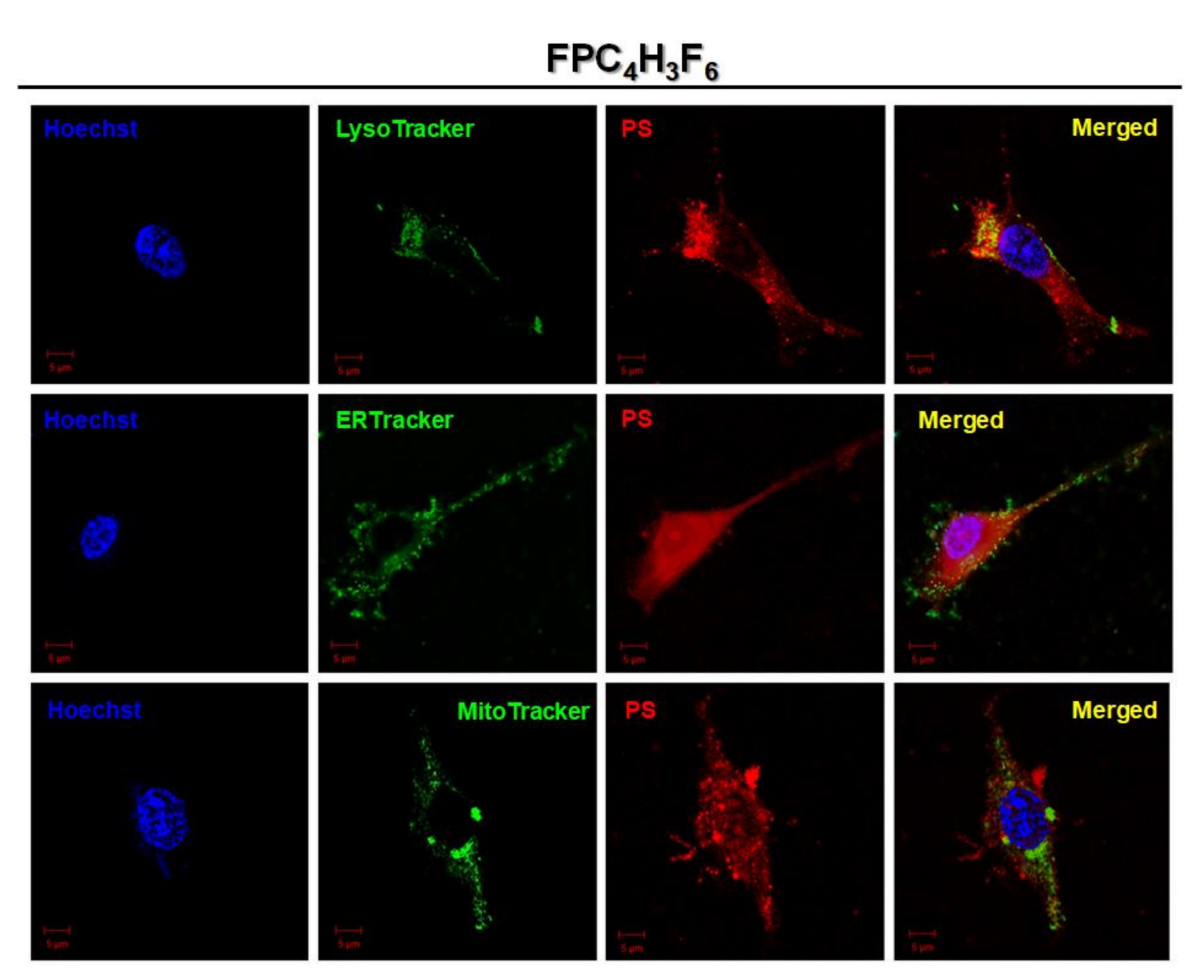
Laser scanning confocal fluorescence microscopy images of cells showing intracellular localization of F_2_POH and FPC_4_H_3_F_6_. Cells were marked with specific probes for endoplasmic reticulum (ERTracker), lysosomes (LysoTracker), and mitochondria (MitoTracker).

The intracellular localization of a photosensitizer is important to determine its initial targets in PDT [50]. We employed confocal laser scanning microscopy (CLSM) to analyze the subcellular accumulation of studied photosensitizers using their intrinsic fluorescence and that of organelle-specific probes. The CT26 cells were cultured for 24 h with FPC_4_H_3_F_6_, taken as representative of sulfonate ester porphyrins, and separately with the hydrophilic derivative (F_2_POH), at 20 μM concentration. For CLSM imaging, each porphyrin was subsequentially incubated with a specific probe for mitochondria (MitoTracker), endoplasmic reticulum (ERTracker), or lysosomes (LysoTracker). The overlaid images are shown in Figure 5, in which the fluorescence of the porphyrin is shown in red, the fluorescence of the organelle-specific probe is shown in green, and blue fluorescence from Hoechst33342 was used to visualize nuclei. The intensity of FPC_4_H_3_F_6_ fluorescence is higher than that of F_2_POH as expected from its higher internalization in the cells. Due to the inconsistency in the labeling pattern on the panels presented for different organelle-probe related to the different imaging planes, the wider field of view for F_2_POH and FPC_4_H_3_F_6_ with more cells in each panel, registered with the same imaging plane is showed in Figure S2. The intracellular fluorescence from FPC_4_H_3_F_6_ presents several distinct red emission pixels and some overlap with both the LysoTracker and the ERTracker, suggesting that structural modification at the sulfonate ester moiety may have taken place after uptake. Interestingly, the fluorescence of F_2_POH also overlaps with these two probes. Sulfonate ester porphyrins likely hydrolyze in the acidic lysosomal environment, and more than one species is present after 24 h of incubation of FPC_4_H_3_F_6_ with the cells. The images registered for the other three photosensitizers are presented in Figures S3–S5. It should be highlighted that these PSs distribute very broadly in various intracellular compartments. A similar effect was also observed for other ester-substituted photosensitizers, e.g., pyropheophorbide *a* methyl ester—which was reported to be localized in the endoplasmic reticulum, Golgi apparatus, lysosomes, and mitochondria in NCI-h446 cells [51].

Based on these data, it can be concluded that the substitution of porphyrin by fluorosulfonate ester increases its uptake by lysosomes and mitochondria, and consequently decreases the localization in the nuclei. To support these data, we include the topographic fluorescence profiles recorded after cells were co-stained with FPC_3_H_7_ or FPC_4_H_3_F_6_ and organelle-specific fluorescent probes as well as Pearson’s correlation coefficients (R), Figure S6. The different localizations of FPC_4_H_3_F_6_ and its hydrolysis products may trigger a greater diversity of mechanisms of cell death [52].

#### 2.4.3. Cytotoxicity in the Dark

**Figure 6:**
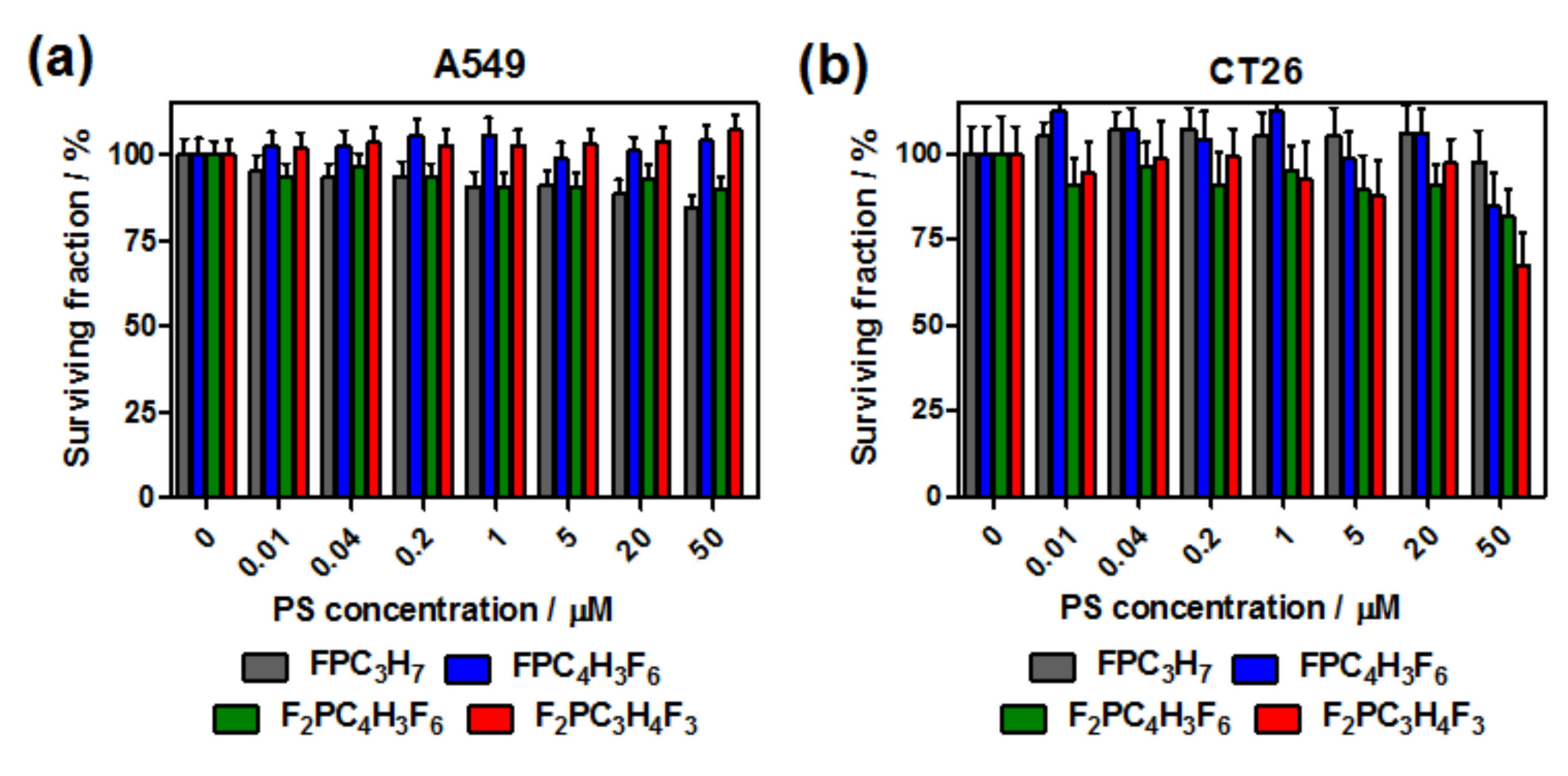
Cytotoxicity of sulfonate ester porphyrins tested in the dark against (**a**) A549 and (**b**) CT26 cancer cells.

To evaluate the potential application of sulfonate ester porphyrins as efficient photodynamic therapeutic agents, their dark and phototoxicity towards A549 and CT26 cells were investigated using MTT assay (Figure 6). The toxicity in the dark of investigated photosensitizers was tested after 24 h of incubation (maximal uptake determined experimentally). Exposure to the cells to concentrations below 50 μM did not reveal significant cytotoxicity. FPC_3_H_7_ at 50 µM showed the highest toxicity towards A549 cells, with ca. 20% mortality. F_2_PC_3_H_4_F_3_ reduced of CT26 cells viability by ca. 40% at the same concentration. Similar results were also obtained for sulfonamide analogs [30,31]. For comparison, sulfonate porphyrins showed cytotoxicity at 100 µM concentrations [29]. The lower dark cytotoxicity of hydrophilic porphyrins may be related to their lower uptake. In view of these low cytotoxicities, we employed a concentration of 20 μM to investigate the *in vitro* efficacy of sulfonate ester photosensitizers.

#### 2.4.4. ROS Generation *In Vitro*

**Figure 7:**
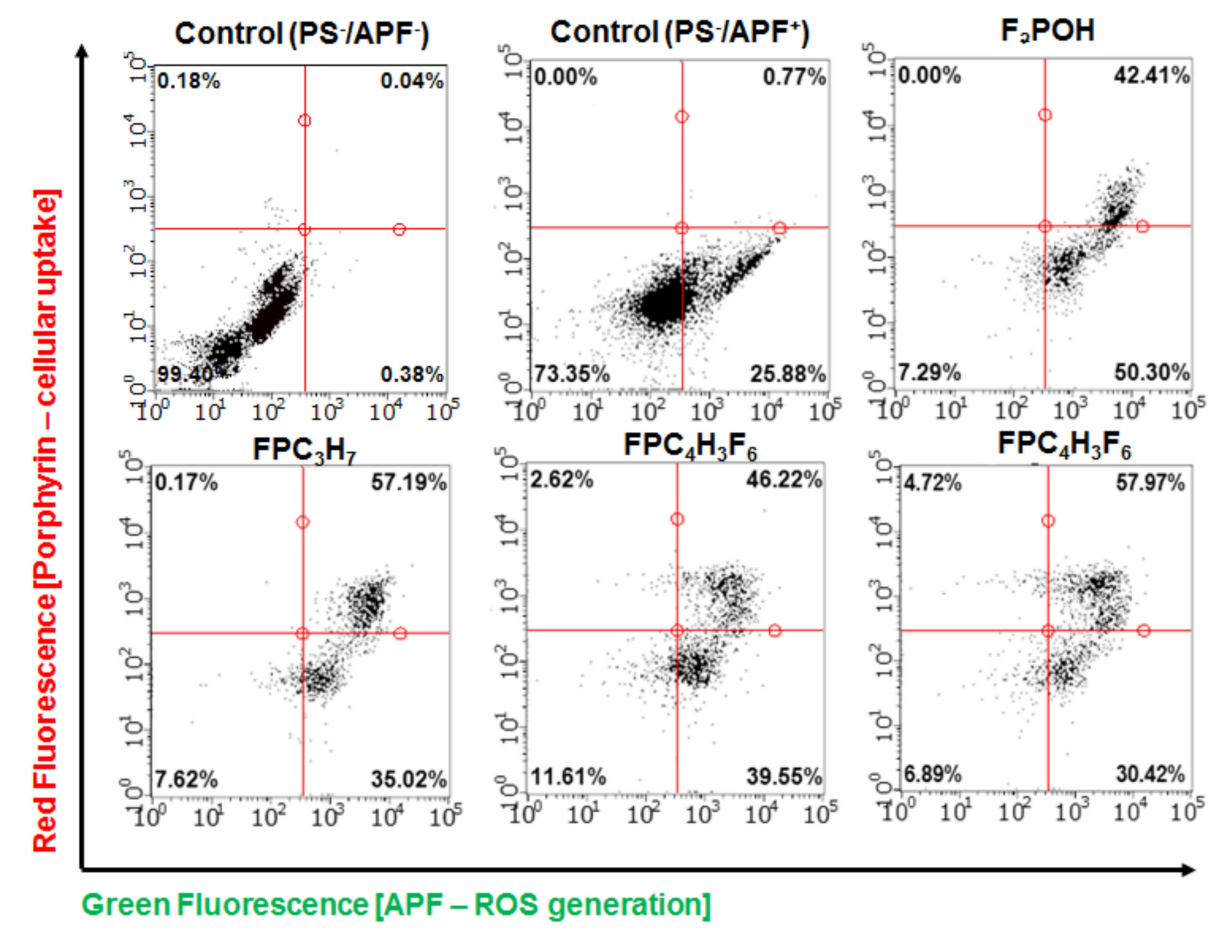
Flow cytometry analysis of reactive oxygen species (ROS) generation *in vitro*. After incubation of the cells with PS (24 h) and APF (2 h), the cells were irradiated. The level of ROS was monitored by the APF fluorescence signal in the green channel and the cellular uptake (red porphyrin fluorescence) was detected as an increased red fluorescence signal.

The ability of the investigated porphyrin to ROS formation *in vitro* was assessed by the flow cytometry. For this purpose, the APF probe (25 µM) was incubated for 2 h following 24 h incubation of CT26 cells with each porphyrin (20 µM). Moreover, due to the fact that singlet oxygen sensor green (SOSG) does not enter living cells, we resorted to use the APF, which also is fluorescent in the green; thus, APF may be used for studying the red fluorophores in the two-color analysis. In contrast to SOSG, the APF is only partially sensitive singlet oxygen but mainly responds to other reactive oxygen species, in particular hydroxyl radicals. Therefore, we ascribed the fluorescence signal to ROS generated by photosensitizers. The results presented in Figure 7 demonstrate that significant green fluorescence appears in the cells upon irradiation with the red light.

Using the total cellular fluorescence as a measure of the amount of ROS formed, the flow cytometry data show that under similar conditions, ROS production is effective for all the photosensitizers accumulated in the cells. These data also demonstrated that the fluorescence intensity of the APF probe in the cells incubated with porphyrin and exposed to 10 J/cm^2^ red light was significantly higher than that of non-stained control cells (PS-/APF-) as well as cells stained only with APF (PS-/APF+), which indicated the detectable signal. The APF-positive population in this sample may be related to the presence of a naturally-occurring intracellular ROS level or APF autofluorescence. The augmentation of the ROS production in cells with accumulated photosensitizers (red fluorescence signal) was nearly three-fold higher than in cells without photosensitizer. Thus, it suggests that the cellular uptake of porphyrins correlates with their enhanced ROS generation *in vitro* and, consequently, the overall photodynamic activity. Moreover, our results indicated that the level of oxidative stress was always higher in cells treated with fluorinated sulfonate ester porphyrins than treated with a sulfonated one (F_2_POH). These studies are also consistent with the data in Figure 3 (cellular uptake) and strengthen the view that the fluorinated sulfoester substituents may increase the phototoxicity of photosensitizers, which correlates best with the efficiency of generation of oxygen-centered radicals.

#### 2.4.5. Photodynamic Effect

**Figure 8:**
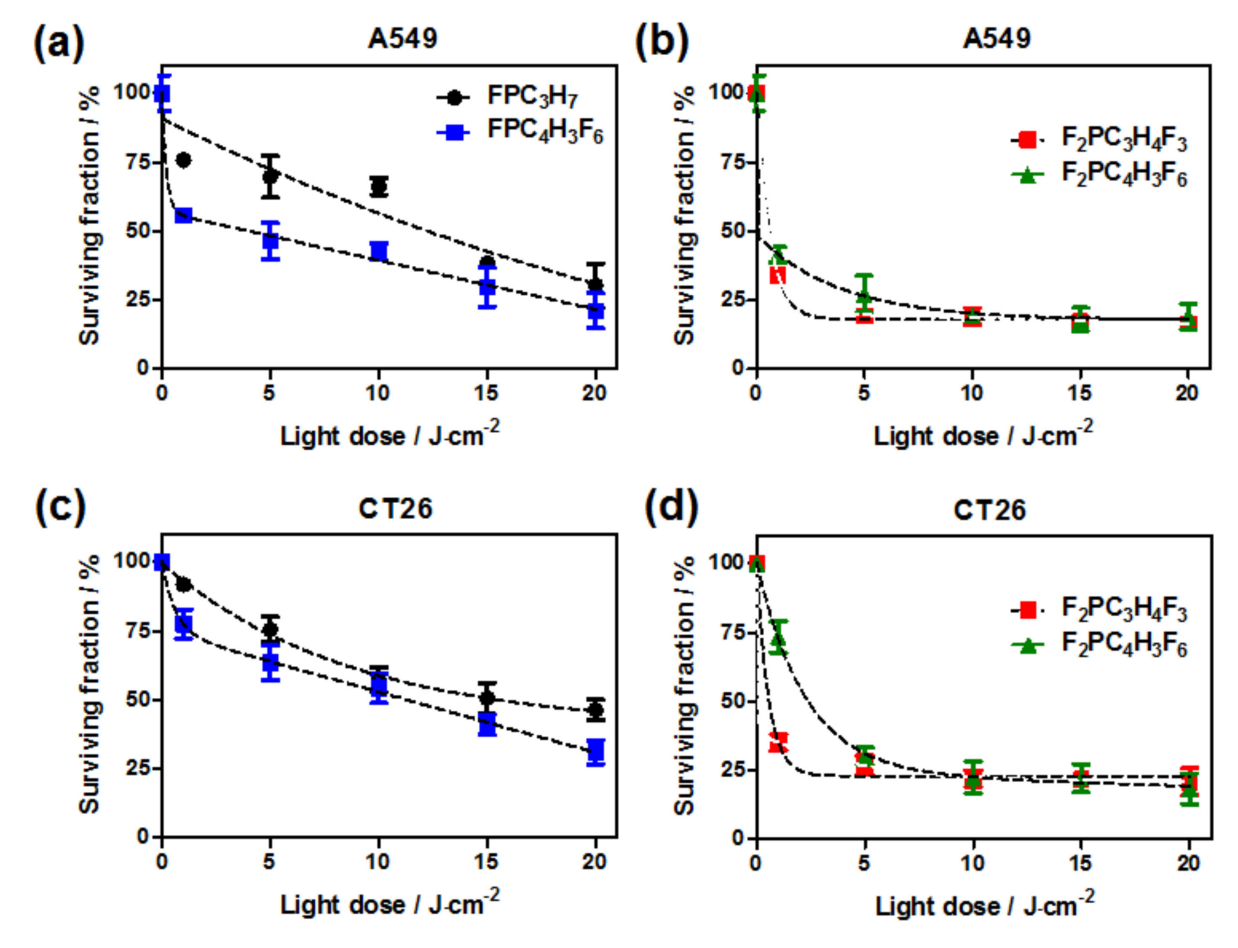
Survival fraction of A549 (**a**,**b**) and CT26 (**c**,**d**) cells in the presence of sulfonate ester porphyrins as a function of applied red-light doses. The data shown are the means ± SEM of three independent experiments.

Cells incubated with photosensitizers were irradiated with various red-light doses. Irradiation of the A549 and CT26 was performed following 24 h cells incubation with 20 μM porphyrins that allow efficient intracellular accumulation (*vide* uptake studies). The survival fraction after the photodynamic effect is presented in Figure 8.

**Figure 9:**
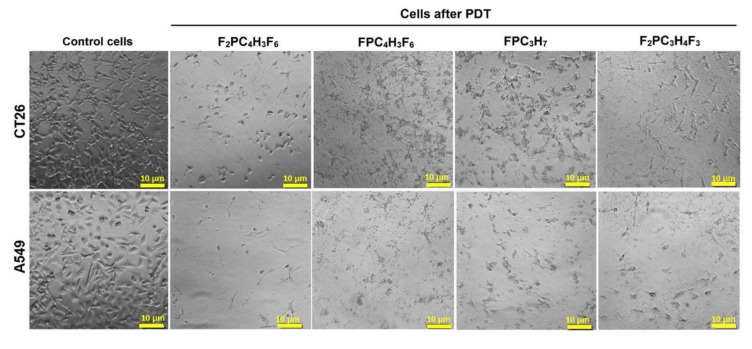
The morphology of A549 and CT26 cells before and 24 h after photodynamic effect with sulfonate ester porphyrins at the drug dose of 20 µM and light dose of 5 J/cm^2^.

The results indicate that both difluoro-substituted porphyrins are more effective in photodynamic killing than monofluorinated derivatives. We found that F_2_PC_3_H_4_F_3_ is particularly phototoxic. Interestingly, this is not the photosensitizer with the highest cellular uptake. However, it is this PS that gives the higher signal with APF and HPF, suggesting that type I reactions may be important for the phototoxicity of this photosensitizer. Moreover, it can be highlighted that sulfonate esters photosensitizers are more phototoxic than the sulfonated one, as expected from the higher intracellular accumulation. To better illustrate the phototoxicity of a group of photosensitizers, we also examined the morphological changes in cells after photodynamic treatment using optical microscopy, see Figure 9. Presented images reveled that all PDT treated cells were partially or completely dead 24 h after treatment.

## 3. Materials and Methods

### 3.1. Chemicals

The 5,10,15,20-tetrakis(2,6-difluoro-3-chlorosulfophenyl) porphyrin alkoxy derivatives were prepared according to reported procedures [6]. All employed solvents were purchased from Sigma-Aldrich (Saint Louis, MO, USA) and used without further purification.

### 3.2. UV/VIS/NIR Electronic Absorption and Emission Spectra Measurements

Solutions containing samples of photosensitizers were dissolved in DMSO. UV/VIS/NIR electronic absorption spectra were recorded in quartz cuvettes (l = 1 cm) using Shimadzu 2100 spectrophotometer (Shimadzu Corp., Kioto, Japan). Fluorescence emission spectra were recorded from 550 nm to 750 nm with excitation at the Soret band (420 nm) using a Fluorescence Spectrometer LS 55 (Perkin Elmer, Cracow, Poland) [31].

### 3.3. Detection of Reactive Oxygen Species

The 3′-p-(aminophenyl)fluorescein (APF), 3′-p-(hydroxyphenyl)fluorescein (HPF), and Singlet Oxygen Sensor Green^®^ (SOSG) were employed as a molecular probe for detection of reactive oxygen species formation during irradiation. Sulfonate ester porphyrin solutions were diluted to a concentration of 5 μM per well in PBS (final DMSO concentration did not exceed 0.5%). Next, the fluorescent probes were added to each well at a final concentration of 10 μM. Photosensitizer solutions were irradiated with the 635 nm or 655 nm laser diode, and the light was delivered for various time intervals. The microplate reader (Tecan Infinite M200 Reader; Tecan, Männedorf, Switzerland) was used for the acquisition of fluorescence signals immediately before and after irradiation [31]. When APF/HPF was employed, fluorescence emission at 515 nm was measured upon excitation at 490 nm. With SOSG, the corresponding values were 525 and 505 nm, respectively.

### 3.4. n-Octanol/Water Partition Coefficients

The n-octanol/PBS partition coefficients were determined following the shake-flask method with minor modification. The porphyrin was dissolved in n-octanol previously saturated with a solution of PBS. The same volume of PBS saturated with n-octanol was added and mixed on a vortex, and then, the phases were separated by centrifugation. Next, the PBS/n-octanol phase was taken and diluted to obtain 0.5% of PBS/n-octanol content in the final solution. This solution was left into the ultrasonic bath. The fluorescence of each solution was measured using Fluorescence Spectrometer LS 55 (Perkin Elmer, Cracow, Poland) and compared with the calibration curve to obtain the concentration of the photosensitizer. The partition coefficient was calculated from the ratio c_oct_/c_PBS_, where c_oct_ and c_PBS_ are the concentrations of the porphyrin derivatives in the n-octanol and the PBS [31].

### 3.5. Cells Culture

Human lung adenocarcinoma (A549) and murine colon carcinoma (CT26) cells were grown in a Dulbecco’s Modified Eagle’s Medium (DMEM) supplemented with 10% fetal bovine serum (FBS) and antibiotics (PAN-Biotech GmbH, Aidenbach, Germany). The cells were cultured in incubators maintained at 37 °C in a 95% atmospheric air and 5% CO_2_ humidified atmosphere. All experiments were performed on cells in the logarithmic phase of growth. Media were replaced every two days, and cells were subcultured using 0.25% trypsin-EDTA.

### 3.6. Cellular Uptake

Cells were seeded on 96-plate microplate (10^4^ per well). After 24 h, the cells were incubated with each porphyrin-based photosensitizer for various time intervals from 2 h up to 24 h. The appropriate controls were included. The solutions of photosensitizers were prepared by diluting the porphyrin stock solution in DMSO with the culture medium to the desired final concentration (20 μM). The highest concentration of DMSO in the medium did not exceed 0.5%. After incubation, the cells were washed two times with warmed PBS and solubilized in 30 μL of Triton X-100 and 70 μL of DMSO/ethanol solution (1:3). The retention of cell-associated porphyrin was detected by fluorescence measurement with the microplate reader (Tecan Infinite M200 Reader, Tecan, Männedorf, Switzerland) [31]. Cellular uptake of investigated photosensitizers was also determined using flow cytometry and quantified based on porphyrin’ red fluorescence. For this analysis, the CT26 cells (0.5×10^6^ cells) seeded in the six-well plate were incubated with each porphyrin at 20 µM for 24 h. After this incubation, cells were washed two times with HBSS and harvested for analysis. Then, cells were collected by centrifugation and then resuspended in 200 μL of PBS. Stained cells were then examined using Guava^®^ easyCyte™ flow cytometer equipped with 488 nm laser. Obtained data were analyzed using InCyte software (MerckMillipore, Burlington, MA, USA).

### 3.7. Confocal Laser Scanning Microscopy (CLSM) Imaging

The intracellular accumulation of selected porphyrins was assessed in the CT26 cancer cell line. Before imaging, CT26 cells were seeded on microscopic slides at a density of 1·10^5^ cells and were kept at 37 °C in a 95% atmospheric air and 5% CO_2_ humidified atmosphere for 24 h. After being washed with fresh medium, the cells were incubated in the dark with 20 μM solution of each porphyrin prepared cell medium for 24 h. Next, after being washed with HBSS, the cells were incubated with specific intracellular organelle probes: 100 nM Mito-Tracker green, 1 µM ERTracker green, 1 μM LysoTracker green (Molecular Probes, Invitrogen Life Technologies; Thermo Fisher Scientific, Waltham, Massachusetts, USA), diluted in HBSS buffer. In addition, cells were incubated for 10 min with Hoechst33342. After 30 min incubation, at 37 °C, in the dark, the cells were washed with HBSS two times, and the slide was transferred to the microscope stage and cells were visualized under a confocal microscope Zeiss LSM 880 (Carl Zeiss, Jena, Germany) with a 63× oil immersion objective. Images were analyzed by Zeiss ZEN Software.

### 3.8. Dark Cytotoxicity and Cells Viability Assay

To assess the dark cytotoxicity of sulfonate ester porphyrins induced in A549 and CT26 cancer cells, after cell attachment, porphyrin solutions prepared in culture medium at concentrations from 0 to 500 μM was added to the cell cultures. Treated cell cultures were incubated for ca. 24 h in the dark. Next, the photosensitizer solutions of each well were removed, cells were washed in PBS, and fresh culture medium supplemented with PBS and antibiotics was added to each well, and cells were returned to the incubator for 24 h. Next, the viability of the cells was calculated based on the MTT (3-(4,5-dimethylthiazol-2-yl)2,5-diphenyl tetrazolium bromide) assay. MTT dissolved in PBS at content 10% of the final solution was added to each well, and the microplates were further incubated for ca. 3 h. The medium was then discarded, and 100 μL of the mixture of DMSO/methanol (1:1) were added to the cultures and mixed thoroughly to dissolve the dark blue crystals of formazan. Formazan quantification was performed using an automatic microplate reader (Tecan Infinite M200 Reader; Tecan, Männedorf, Switzerland) by absorbance measurements with a 565 nm test wavelength.

### 3.9. ROS Generation In Vitro

ROS generation *in vitro* was investigated using dual-color flow cytometry analysis quantified after 24 h incubation of CT26 cells with each photosensitizer (20 µM). After this incubation, cells were washed with HBSS and then incubated with APF (25 µM) prepared in HBSS for the next 2 h. After this time, cells were washed twice with HBSS and irradiated with 10 J/cm^2^ red light (LED diode, 635 ± 20 nm, Instytut Fotonowy, Cracow, Poland). After irradiation, cells were collected by centrifugation, resuspended in 200 μL of HBSS examined using Guava^®^ easyCyte™ flow cytometer equipped with 488 nm laser. Obtained data were analyzed using InCyte software (Merck Millipore, Burlington, MA, USA).

### 3.10. In Vitro Phototoxicity of Sulfonate Ester Porphyrins

The A549 and CT26 cells were seeded into a 96-wells culture plate (10^4^ cells per well) and kept at 37 °C in a 95% atmospheric air and 5% CO_2_ humidified atmosphere for 24h. Cells were incubated with fluorinated sulfonate ester porphyrins (F_2_PC_3_H_4_F_3_, FPC_4_H_3_F_6_, FPC_3_H_7_, and F_2_PC_3_H_4_F_6_) at 20 μM for 24 h. The photosensitizer’s solutions were then removed, the cells were washed in PBS and irradiated with the 635 nm laser diode light (High Power Monochromatic Light System and LED Illuminator, Instytut Fotonowy, Poland) or 655 nm laser light for various time intervals. In all experiments, the light dosimetry was performed and controlled using Ophir NOVA II radiometer (Laser Measurements Group, Ophir Optronics, Jerusalem, Israel). After the irradiation, the PBS was replaced by the growth medium, and plates were returned to the incubator (37 °C, 5% CO_2_). Twenty-four hours after irradiation, cells viability was determined by an MTT assay. Data were expressed as mean fluorescence intensity value and standard error of the mean. The morphology of A549 and CT26 cells were investigated before and after photodynamic treatment. The cells were observed using optical and fluorescence microscopy (Olympus BX51 Microscope; Olympus, Tokio, Japan).

## 4. Conclusions

The design and development of new photosensitizers for PDT remains an appealing research field. In this work, the properties of sulfonate ester fluorinated porphyrins were examined to investigate the potential of this molecular template for PDT. Their absorption bands at 630–650 nm do not have particularly high absorption coefficients, but if this template is useful for PDT, they may, in a later stage, be reduced to the corresponding chlorins or bacteriochlorins. We showed that their triplet lifetimes are long enough and lead to ROS quantum yields higher than 0.6. It has been found that the biological properties of photosensitizers depend greatly on the molecular substitution pattern. Compared to the hydrophilic analog (F_2_POH), the sulfonate esters indicate higher lipophilicity as well as more efficient ROS generation. *In vitro* studies of cellular uptake and subcellular localization showed important differences between studied photosensitizers. It seems that the sulfonate ester fluorinated porphyrins are efficiently internalized by the cells and then may hydrolyze to some extent. The sulfonate ester porphyrins were tested against two cancer cell lines of different tissue origin, A549 (human lung adenocarcinoma) and CT26 (murine colon carcinoma). Cytotoxicity assays indicated that the onset of cytotoxicity in the dark takes place at ca. 50 µM, which is a rather low cytotoxicity. One of the sulfonate ester fluorinated porphyrins (F_2_PC_3_H_4_F_3_) exhibited remarkable phototoxicity. This result is enabled by the low tendency to aggregate, high efficiency in generating ROS, and improved cellular uptake comparing to sulfonated porphyrin. However, it should be clearly stated that its highest photodynamic efficiency among the tested compounds does not correlate best with either PS cellular uptake or singlet oxygen quantum yield but with the efficacy of this PS to generate oxygen-centered radicals *via* type I mechanism. Fluorinated porphyrins with sulfonate ester substituents also bearing fluorine atoms are promising candidates for new templates of PDT photosensitizers.

## Figures and Tables

**Table 1:** Values of n-octanol/water partition coefficients determined for sulfonated and sulfonate ester halogenated porphyrin derivatives.

Photosensitizer	logP_OW_
F_2_POH	−1.7
F_2_PC_3_H_4_F_3_	1.4
FPC_4_H_3_F_6_	1.6
FPC_3_H_7_	1.6
F_2_PC_4_H_3_F_6_	1.5

## Supplementary Materials

Supplementary materials can be found at https://www.mdpi.com/1422-0067/21/8/2786/s1.
