# Supplementary material for: Enhanced Cellular Uptake and Photodynamic Effect with Amphiphilic Fluorinated Porphyrins: The Role of Sulfoester Groups and the Nature of Reactive Oxygen Species
Source: Int J Mol Sci. 2020 Apr 16;21(8):2786. doi: 10.3390/ijms21082786 (PMC7216003; doi:10.3390/ijms21082786)
Supplement: Supplementary file 1 [file ijms-21-02786-s001.pdf]

Supporting Information for

Enhanced cellular uptake and photodynamic effect with amphiphilic fluorinated porphyrins: the role of sulfoester groups and the nature of reactive oxygen species

Barbara Pucelik[1,2], Adam Sułek[1], Agnieszka Drozd[1], Grażyna Stochel[1], Mariette M. Pereira[3], Sara M. A. Pinto[3], Luis G. Arnaut[3] and Janusz M. Dąbrowski[1*]

[1] Faculty of Chemistry, Jagiellonian University, Krakow, Poland
[2]Małopolska Center of Biotechnology, Jagiellonian University, Krakow, Poland
[3]Chemistry Department, University of Coimbra, Portugal
* Corresponding author. Tel.: +48-12-686-2488; fax: +48-12-686-2750, e-mail address: jdabrows@chemia.uj.edu.pl (J.M.D)

1. *Theoretical studies*

The first step of computational studies was optimizations of geometry by density functional theory using Gaussian 09 code. Electronic ground state and structures optimization has been done by using the M06 exchange and correlation functional coupled with the 6-31G* basis set. Electronic absorption spectra and singlet-triplet energy gaps have been computed using the B3LYP exchange-correlation functionals. TDDFT calculations have been performed in a vacuum and in ethanol with PCM model. M06/6-31+G*, and B3LYP/6-31+G* have been chosen.

Table S1. M06/6-31+G* main vertical singlet excitation energies ΔE (eV), oscillator strengths *f*, and MO contribution to the transitions (%), comparison with experimental spectrum in ethanol. The continuum solvation model (ethanol) was used for $F_2PC_3H_4F_3$, $F_2PC_4H_3F_6$, $FPC_4H_3F_6$ and $F_2PC_3H_7$.

Compound	Excited state	ΔE (eV)	ΔE_{exp} (eV)	f	MO contribution (>10%)
$F_2PC_3H_4F_3$	$S_1(Q_x)$	2.21	1.91	0.00	**H→L+1 58%**, H-1→L 40%
	$S_2(Q_y)$	2.35	2.44	0.02	**H→L 56%**, H-1→L+1 42%
	$S_3(B_x)$	3.09	3.00	1.17	**H-1→L 50%,** H→L+1 32%, H-3→L+1 16%,
	$S_4(B_y)$	3.19		1.67	**H-1→L+1 54%**, H→L 40%
	T_1	1.46		0.00	**H→L 76%**, H-1→L+1 24%
	T_2	1.79		0.00	**H→L+1 82%**, H-1→L 14%
	T_3	1.96		0.00	**H-1→L+1 58%**, H-1→L 19%, H→L 19%
$F_2PC_4H_3F_6$	$S_1(Q_x)$	2.21	1.91	0.00	**H-1→L 50%**, H→L+1 35%
	$S_2(Q_y)$	2.36	2.43	0.00	**H→L 46%**, H-1→L 39%
	$S_3(B_x)$	3.03	2.98	0.79	**H→L+1 41%**, H-3→L 28%, H-1→L 25%
	$S_4(B_y)$	3.14		1.26	**H-1→L+1 43%,** H→L 34%
	T_1	1.50		0.00	**H-1→L 67%**, H→L+1 16%, H→L 12%
	T_2	1.79		0.00	

	T_3	1.93		0.00	**H→L 50%**, H-1→L+1 36%
$FPC_4H_3F_6$	$S_1(Q_x)$	2.21	1.93	0.01	**H→L 60%**, H-1→L+1 39%
	$S_2(Q_y)$	2.35	2.43	0.00	**H-1→L 52%**, H→L+1 48%
	$S_3(B_x)$	3.03	3.00	0.87	**H-1→L+1 46%**, H→L 25%, H-3→L 23%
	$S_4(B_y)$	3.12		1.31	**H→L+1 45%**, H→L+1 38%
	T_1	1.50		0.00	**H→L+1 64%**, H-1→L 36%
	T_2	1.76		0.00	**H→L 88%**, H-1→L+1 11%
	T_3	1.93		0.00	**H-1→L 64%**, H→L+1 36%
FPC_3H_7	$S_1(Q_x)$	2.22	1.92	0.00	**H→L 50%**, H-1→L+1 35%
	$S_2(Q_y)$	2.36	2.43	0.00	**H-1→L 46%**, H→L 38%
	$S_3(B_x)$	3.03	2.98	0.81	**H-1→L+1 32%**, H-3→L 28%, H→L 19%, H→L+1 11%
	$S_4(B_y)$	3.15		1.33	**H→L+1 37%**, H-1→L 29%, H-1→L+1 11%
	T_1	1.50		0.00	**H→L+1 50%**, H-1→L 35%
	T_2	1.79		0.00	**H→L 70%**, H-1→L 15%, H-1→L+1 10%
	T_3	1.93		0.00	**H→L 50%**, H→L+1 32%

Table S2. M06/6-31+G* main vertical singlet excitation energies OE (eV), oscillator strengths *f*, and MO contribution to the transitions (%), comparison with experimental spectrum in vacuum.

Compound	Excited state	ΔE (eV)	f	ΔE_{exp} (eV)	MO contribution (>10%)
$F_2PC_3H_4F_3$	$S_1(Q_x)$	2.21	0.00	1.91	**H→L+1 58%**, H-1→L 40%
	$S_2(Q_y)$	2.35	0.02	2.44	**H→L 56%**, H-1→L+1 42%
	$S_3(B_x)$	3.09	1.17	3.00	**H-1→L 50%,** H→L+1 32%, H-3→L+1 16%,
	$S_4(B_y)$	3.19	1.67		**H-1→L+1 54%**, H→L 40%
	T_1	1.46	0.00		**H→L 76%**, H-1→L+1 24%
	T_2	1.79	0.00		**H→L+1 82%**, H-1→L 14%
	T_3	1.96	0.00		**H-1→L+1 58%**, H-1→L 19%, H→L 19%
$F_2PC_4H_3F_6$	$S_1(Q_x)$	2.21	0.00	1.91	**H→L+1 57%**, H-1→L 41%
	$S_2(Q_y)$	2.35	0.02	2.43	**H→L 57%**, H-1→L+1 41%,
	$S_3(B_x)$	3.15	0.79	2.98	**H-1→L 47%**, H→L+1 27%, H-3→L 26%
	$S_4(B_y)$	3.28	1.15		**H-2→L+1 89%**
	T_1	1.44	0.00		**H→L 76%**, H-1→L+1 23%
	T_2	1.80	0.00		**H→L+1 82%**, H-1→L 16%
	T_3	1.95	0.00		**H-1→L 84%**, H→L+1 15%
$FPC_4H_3F_6$	$S_1(Q_x)$	2.20	0.00	1.93	**H→L+1 55%**, H-1→L 40%
	$S_2(Q_y)$	2.34	0.02	2.43	**H→L 55%**, H-1→L+1 40%
	$S_3(B_x)$	3.14	0.79	3.00	**H-1→L 45%**, H→L+1 23%, H-3→L 21%
	$S_4(B_y)$	3.28	1.15		**H-1→L+1 59%**, H-1→L 36%

	T_1	1.45	0.00		**H→L 73%**, H-1→L+1 25%
	T_2	1.75	0.00		**H→L+1 83%**, H-1→L 15%
	T_3	1.99	0.00		**H-1→L+1 70%**, H→L+1 25%
F_2PCH_3	$S_1(Q_x)$	2.20	0.02	1.92	**H→L+1 56%**, H-1→L 34%
	$S_2(Q_y)$	2.34	0.03	2.43	**H→L 55%**, H-1→L+1 36%
	$S_3(B_x)$	3.10	1.19	2.98	**H-1→L 45%**, H→L+1 25%, H-1→L 12%
	$S_4(B_y)$	3.28	1.72		H-1→L 10% **H-1→L+1 49%,** H→L 32%
	T_1	1.45	0.00		**H→L 74%**, H-1→L+1 18%
	T_2	1.75	0.00		**H→L+1 85%**
	T_3	1.98	0.00		**H-1→L+1 69%**, H→L 16%, H-1→L 11%

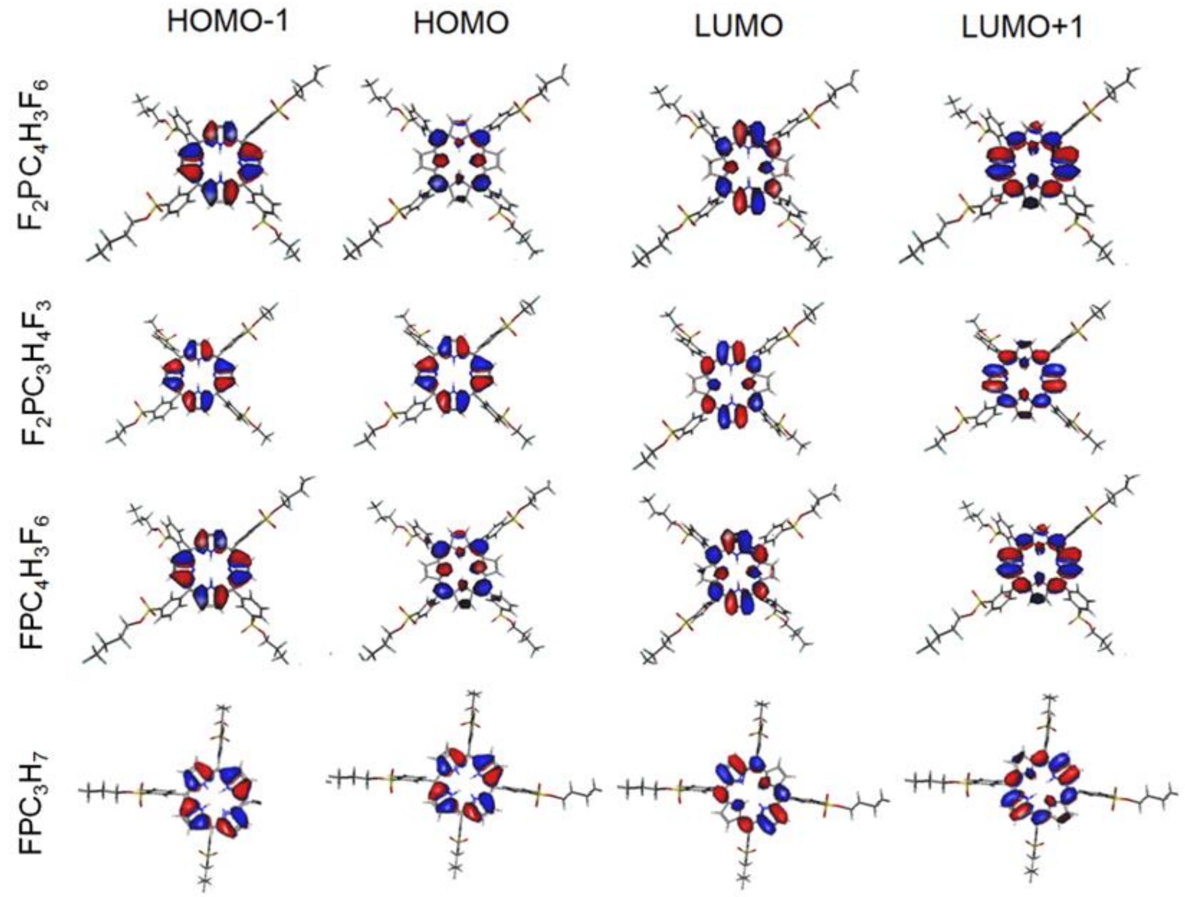

Figure S1. Isodensity contour plots for investigated porphyrin derivatives. The colors reflect the phases of the orbitals. Thus, the blue color reflects a positive phase, whereas the red color refers to a negative phase.

2. CLSM imaging and subcellular localization

Due to the show a wider field of view for both photosensitizers with more cells in each panel, registered with the same imaging plane. We also checked the localization in the nuclei and performed the

colocalization analysis for Hoechst (blue) and porphyrin (red) with Pearson's correlation coefficient (R) expressing the intensity correlation of colocalizing objects in each component of a dual-color image. The result for F_2POH is 0.506, which suggests a good correlation, but for $FPC_4H_3F_6$ we indicated R= -0.073, and consequently, no correlation. This confirmed relatively good localization of F_2POH in the nuclei.

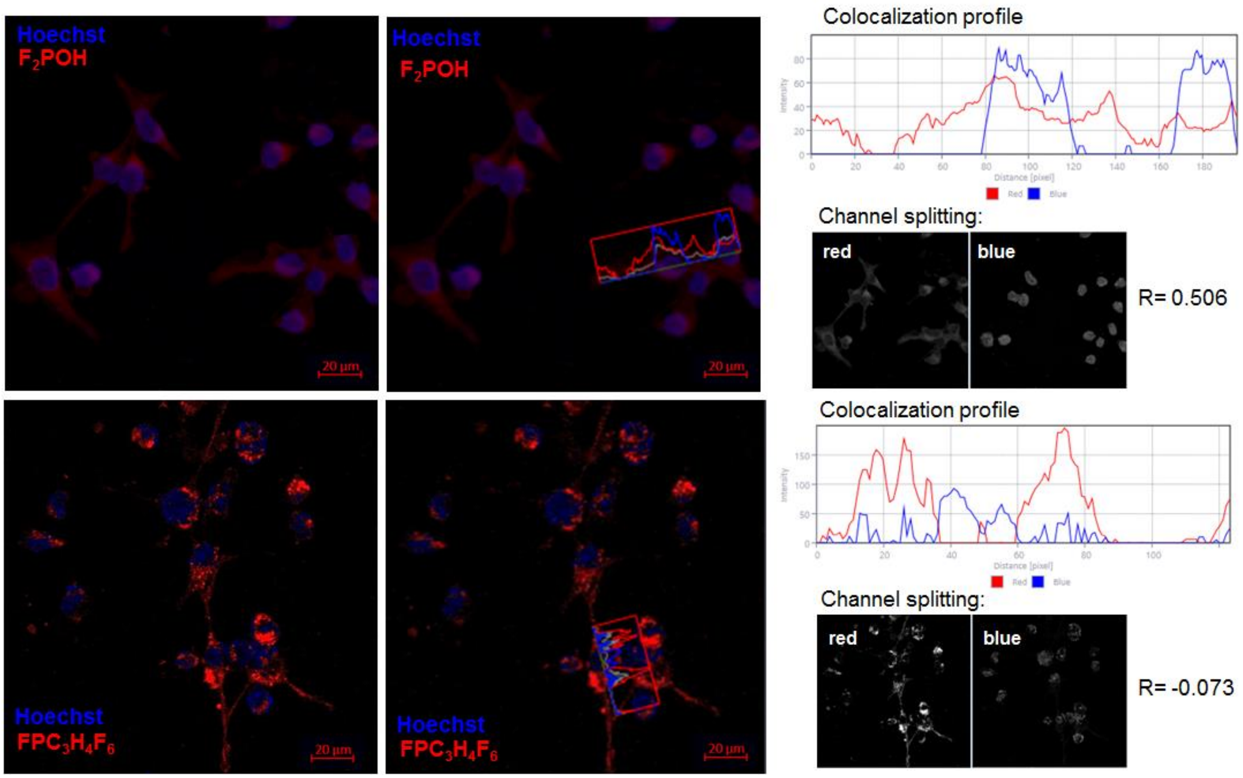

Figure S2. CT26 cells distribution incubated with F_2POH and $FPC_4H_3F_6$ registered with the same imaging plane (left) and their colocalization profiles (with Hoechst33342) with channel splitting and calculated Pearson's coefficients (R) (right).

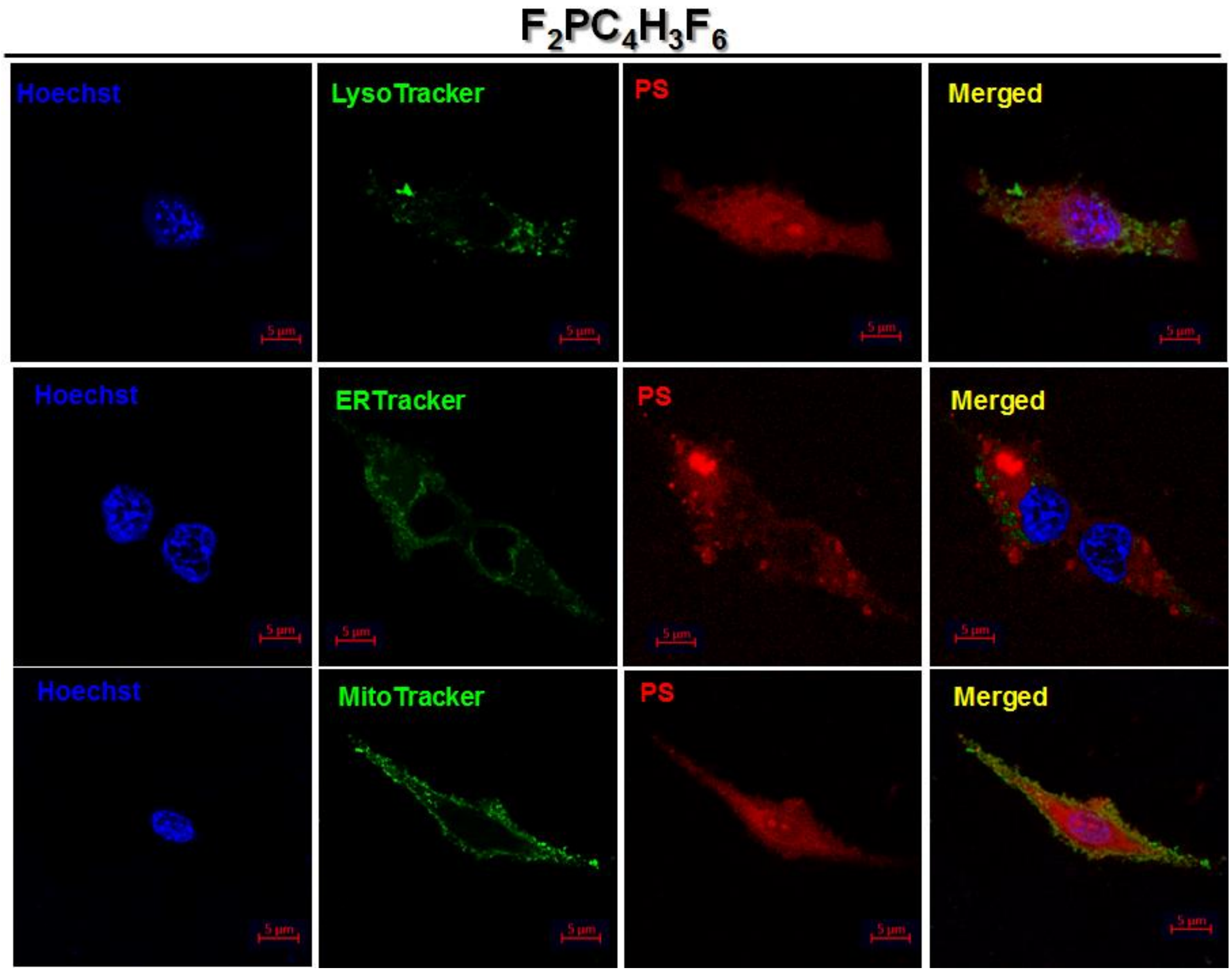

Figure S3. Laser scanning confocal fluorescence microscopy images of cells showing intracellular localization of $F_2PC_4H_3F_6$. Cells were marked with specific probes for endoplasmic reticulum (ER-Tracker), lysosomes (Lyso-Tracker), and mitochondria (Mito-Tracker).

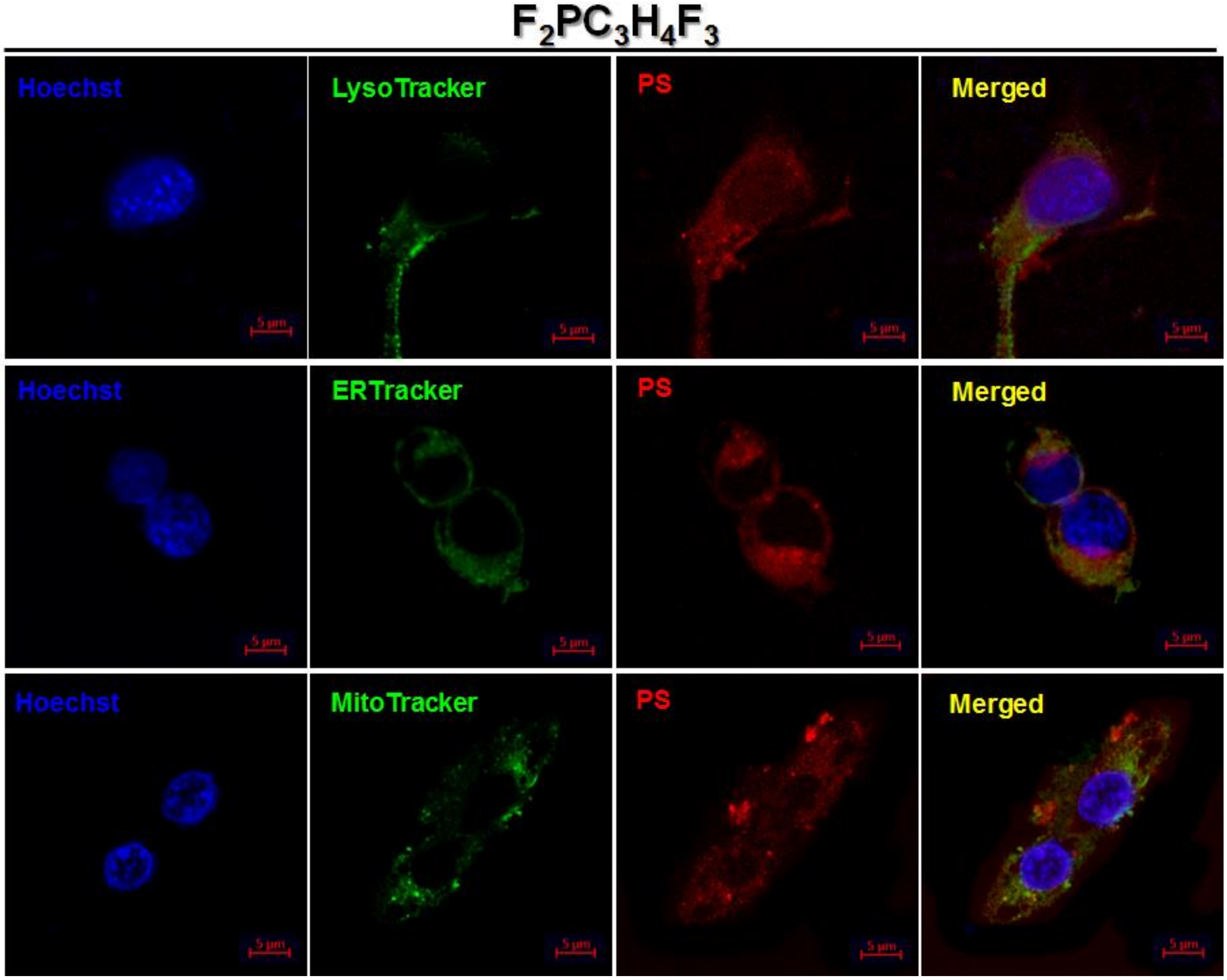

Figure S4. Laser scanning confocal fluorescence microscopy images of cells showing intracellular localization of $F_2PC_3H_4F_3$. Cells were marked with specific probes for endoplasmic reticulum (ER-Tracker), lysosomes (Lyso-Tracker), and mitochondria (Mito-Tracker).

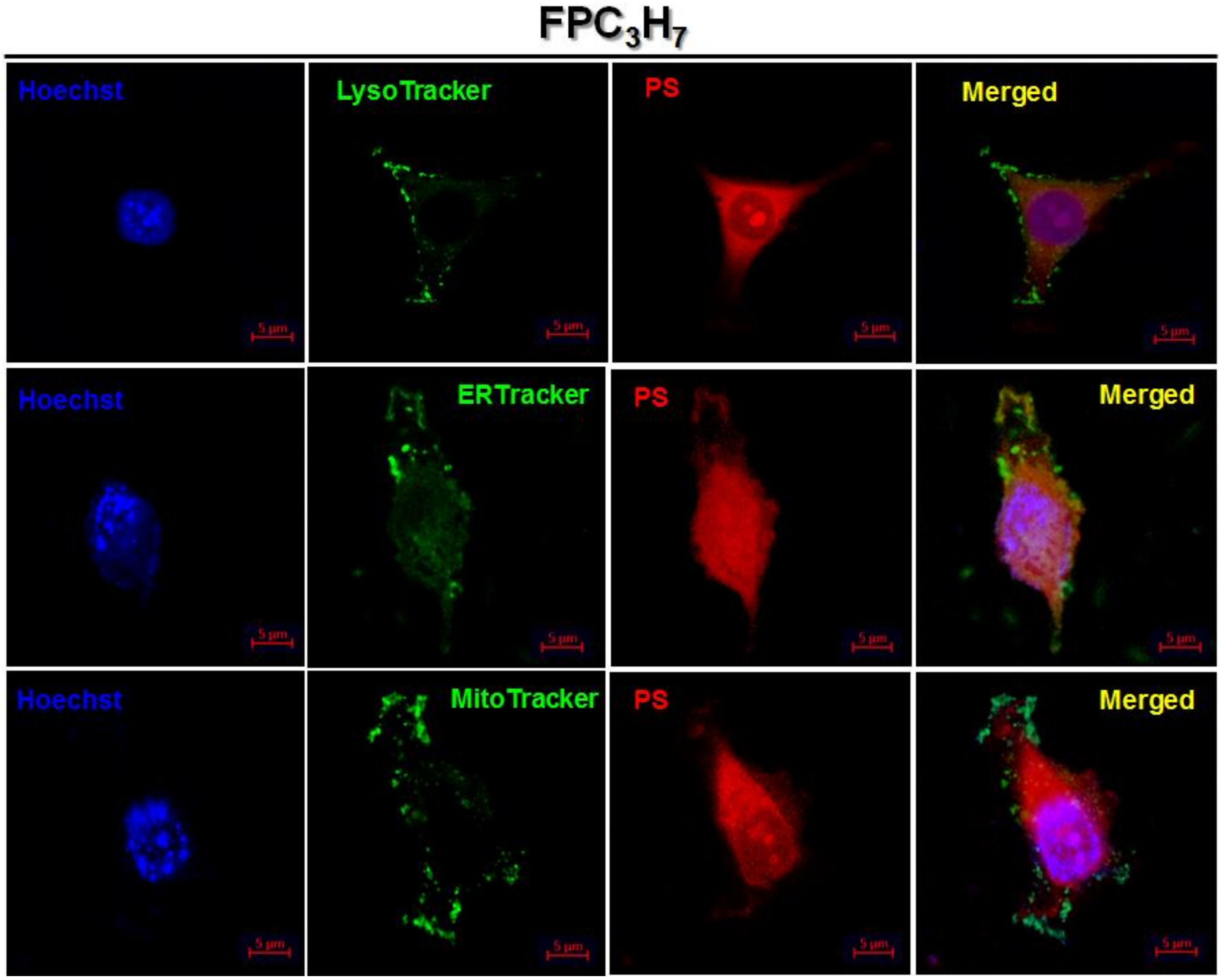

Figure S5. Laser scanning confocal fluorescence microscopy images of cells showing intracellular localization of FPC_3H_7. Cells were marked with specific probes for endoplasmic reticulum (ER-Tracker), lysosomes (Lyso-Tracker), and mitochondria (Mito-Tracker).

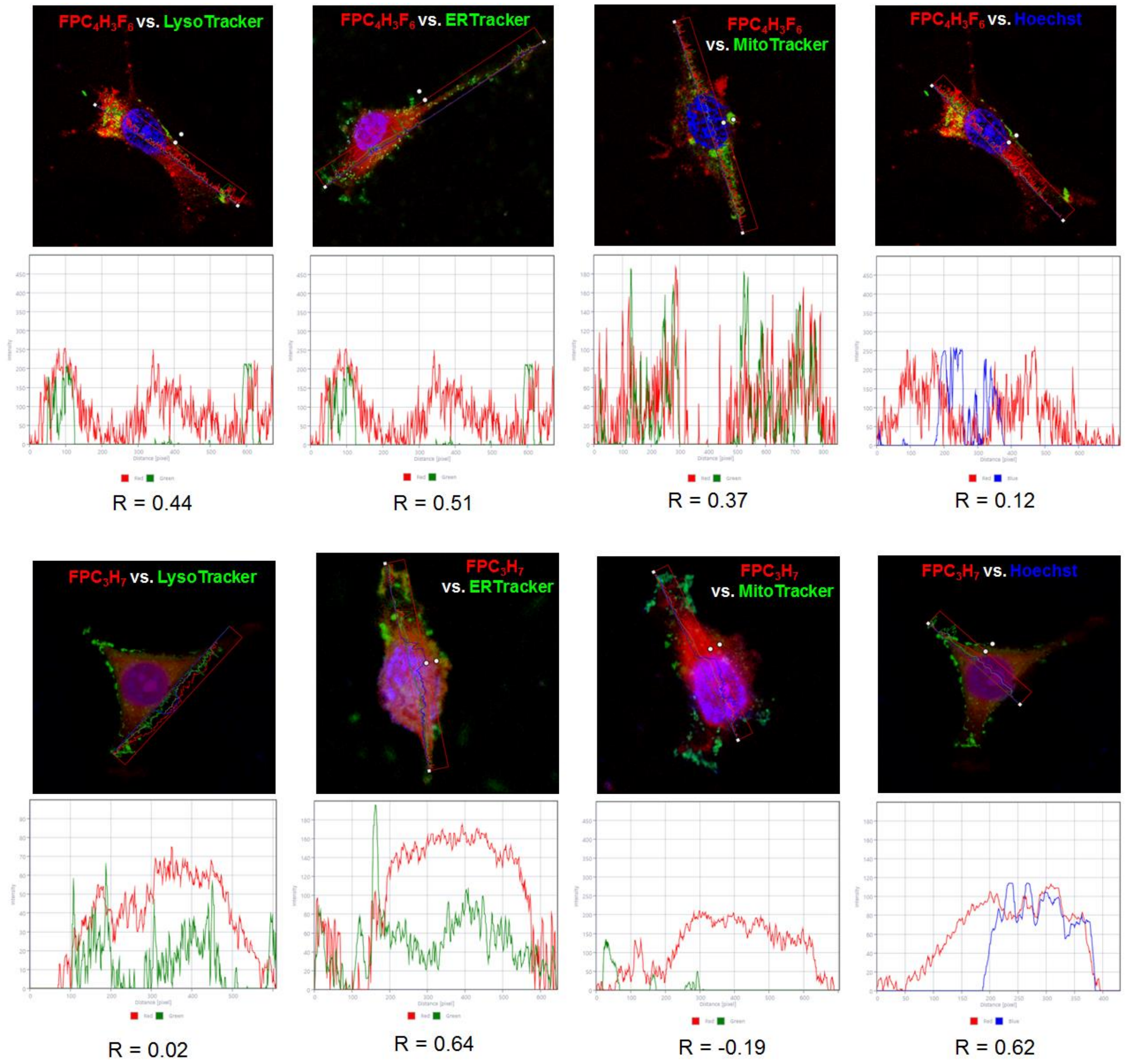

Figure S6. The topographic fluorescence profiles recorded after cells co-stained with FPC_3H_7 or $FPC_4H_3F_6$ and organelle-specific fluorescent probes as well as Pearson's correlation coefficients (R)
